# FOSTERING INTERPROFESSIONAL COLLABORATION IN HISPANIC GERIATRIC PATIENT CARE: NEW GME PROGRAM AND PALLIATIVE TEAM

**DOI:** 10.1093/geroni/igz038.1086

**Published:** 2019-11-08

**Authors:** Jessica G Martin, Andrya Rivera-Burciaga, Cesar Gutierrez, Andrew Dentino, Vianis Bravo

**Affiliations:** 1 University of Texas Rio Grande Valley, Edinburg, Texas, United States; 2 DHR Health, Edinburg, Texas, United States

## Abstract

Hispanic minorities have a higher incidence of chronic disease which may result in increased hospitalizations and life-threatening illness. Our growing geriatric population led our community hospital to create a dedicated Palliative Care department, an interprofessional team of physicians, nursing, pharmacy, social work, counselors, and chaplains whose collaborative practice has improved outcomes thus strengthening healthcare delivery. When our new medical school established graduate programs, including Internal Medicine residency and Hospice/Palliative Medicine fellowship, our team embraced the opportunity to optimize our palliative service through enhanced interprofessional care. We created a geriatric rotation on July 1, 2018 in which 60% of residents worked with palliative care professionals as a consult team bringing together the inpatient resident service and palliative interdisciplinary team. This collaborative model allowed the palliative team to interface with our trainees and teach them to identify the range of needs of older adults early on in their care. Residents reported 100% satisfaction on evaluations, specifically on clinical training, goal fulfillment, and team support. Our learners valued the opportunity to learn with and from other healthcare professionals. Supervising providers also felt that working with residents was beneficial to their practice habits (i.e., providing evidence-based practices, application of guidelines), which offered them a more holistic approach in caring for patients and families. The interprofessional collaboration between a community hospital and medical school to educate and train clinicians who care for individuals with advanced illness has fostered confidence, trust, mutual respect, open communication and effective sharing of critical information for both clinicians and patients.

